# Hedonic appreciation and verbal description of pleasant and unpleasant odors in untrained, trainee cooks, flavorists, and perfumers

**DOI:** 10.3389/fpsyg.2014.00012

**Published:** 2014-01-24

**Authors:** Caroline Sezille, Arnaud Fournel, Catherine Rouby, Fanny Rinck, Moustafa Bensafi

**Affiliations:** ^1^CNRS, UMR5292, INSERM1028, Lyon Neuroscience Research Center, University of LyonLyon, France; ^2^Lidilem Laboratory, University of GrenobleGrenoble, France

**Keywords:** olfaction, expertise, hedonic, emotion, perfumery

## Abstract

Olfaction is characterized by a salient hedonic dimension. Previous studies have shown that these affective responses to odors are modulated by physicochemical, physiological, and cognitive factors. The present study examined expertise influenced processing of pleasant and unpleasant odors on both perceptual and verbal levels. For this, performance on two olfactory tasks was compared between novices, trainee cooks, and experts (perfumers and flavorists): Members of all groups rated the intensity and pleasantness of pleasant and unpleasant odors (perceptual tasks). They were also asked to describe each of the 20 odorants as precisely as possible (verbal description task). On a perceptual level, results revealed that there were no group-related differences in hedonic ratings for unpleasant and pleasant odors. On a verbal level, descriptions of smells were richer (e.g., chemical, olfactory qualities, and olfactory sources terms) and did not refer to pleasantness in experts compared to untrained subjects who used terms referring to odor sources (e.g., candy) accompanied by terms referring to odor hedonics. In conclusion, the present study suggests that as novices, experts are able to perceptually discriminate odors on the basis of their pleasantness. However, on a semantic level, they conceptualize odors differently, being inclined to avoid any reference to odor hedonics.

## INTRODUCTION

Hedonic treatment is a crucial level of processing sensory information. The sense of smell is of particular interest in this regard: in humans, odors induce attractive or repulsive reactions and may influence cognition and behavior in various contexts (Alaoui-Ismaili et al., 1997a,b; Yeshurun and Sobel, 2010; Croy et al., 2011). From a cognitive point of view, odor-grouping experiments showed that hedonics is the most salient dimension of olfaction (Harper, 1966; Berglund et al., 1973; Schiffman, 1977). In these studies, subjects were exposed to various pairs of olfactory stimuli and asked to judge their similarity. It was usually observed that two main clusters were formed: one grouping together pleasant and the other unpleasant odors (Schiffman, 1974; Godinot et al., 1995).

Whereas psychophysical investigations have shown that such hedonic processing of smells is influenced by physicochemical properties (Khan et al., 2007; Mandairon et al., 2009; Poncelet et al., 2010; Joussain et al., 2011; Kermen et al., 2011; Zarzo, 2011), many other experiments, however, showed that odor pleasantness can be modulated by physiological (Fernandez et al., 2013; Joussain et al., 2013a,b) or cognitive factors (Herz, 2003; Rolls, 2004; de Araujo et al., 2005; Barkat et al., 2008; Rinck et al., 2011). For example, it has been shown that pleasantness judgments are enhanced when subjects are able to identify the odorant source (Ayabe-Kanamura et al., 1998). When verbal information about an odor is available, subjects shift their pleasantness judgment in line with the affective connotation of the label (Herz, 2003). Such top-down modulation by verbal association has been found even in children (Bensafi et al., 2007; Rinck et al., 2011). In summary, it would seem that both bottom-up (molecular feature coding) and top-down (training and language) processes contribute to build our hedonic responses to smells, which may be thus very variable across individuals.

Another factor that may explain olfactory individual differences is expertise. Training and verbal associations are crucial in professional situations in which odorants have to be associated systematically to label in order to ensure a common vocabulary to enhance perceptual agreement between individuals. Past and more recent studies showed that experts in olfaction used more consistent, rich, and precise language to describe smells (Bende and Nordin, 1997; Valentin and Chollet, 2000; Parr et al., 2002). Moreover, it has been shown that wine experts use more specific and relevant wine descriptors (Zucco et al., 2011). Although experts are known not only to acquire a systematic knowledge of the chemistry of odorants but also to learn to describe olfactory qualities of odorants and odor sources in a shared language, very little is known about the importance of hedonic processing in both the ways: (i) they describe but also (ii) they perceive smells. On a descriptive level, the literature in the field suggests that whereas pleasantness is a prominent attribute that drives odor verbalizations (Dubois and Rouby, 1997; Dubois, 2000), experts may be inclined to avoid any reference to pleasantness (Yoshida, 1964; Ehrlichman and Bastone, 1992; Holley, 2002). In the present study, we aim to test experimentally this hypothesis on a verbal level and to further assess how expertise modulates hedonic perception of odors. To this end, experts and non-experts in olfaction were compared during two olfactory tasks: (i) a verbal description task whereby participants were asked to freely describe odors and (ii) a perceptual rating task whereby participants were asked to judge the pleasantness of odors. Practically, four groups of subjects, differing in their levels of expertise, were tested: (i) an untrained group, (ii) a group of apprentice cooks, who had no specific course on olfaction but were daily exposed to odors, (iii) a group of experts in aroma formulation, and (iv) a group of experts in perfume formulation.

Moreover, because there is evidence of the existence of two different systems dedicated to treating aversive and appetitive smells [unpleasant odors are processed faster than pleasant ones (Bensafi et al., 2002d; Jacob et al., 2003), induced specific patterns of autonomic (Miltner et al., 1994; Brauchli et al., 1995; Ehrlichman et al., 1995; Alaoui-Ismaili et al., 1997a,b; Ehrlichman et al., 1997; Bensafi et al., 2002a,c) and olfactomotor responses (Bensafi et al., 2003a; Rouby et al., 2009) and specific neural activations (Zald and Pardo, 1997; Gottfried et al., 2002b; Anderson et al., 2003; Rolls et al., 2003; Royet et al., 2003; Bensafi et al., 2012)], odor hedonic valence *per se* was included as a factor in the analysis. Here, all participants were thus presented with unpleasant and pleasant odorant molecules. Specific hypotheses were: (i) on a verbal and descriptive level, experts (flavorists and perfumers) should use precise terminology without reference to pleasantness, whereas non-experts (novices and trainee cooks) should use less precise terminology accompanied by references to pleasantness; (ii) on a perceptual level, experts should not consider pleasantness and thus should rate pleasant odors as less pleasant and unpleasant odors as less unpleasant than non-experts.

## MATERIALS AND METHODS

### SUBJECTS

Sixty-four subjects without neurological disease or olfactory disorder were tested. Participants were divided into four groups according to their level of expertise: (i) a group of untrained individuals (“novices”: *n* = 16; mean age, 23.5 ± 0.423 years; six male), composed of subjects who had no specific training on olfaction; (ii) a group of trainee cooks (“trainee cooks”: *n* = 16; mean age, 21.313 ± 0.285 years; nine male), composed of subjects in their second year of training in a cookery institute where they received no specific training in olfaction, but were exposed daily to odors; (iii) a group of flavorists (“flavorists”: *n* = 16; mean age, 31.063 ± 2.765 years; three male; with 9.065 ± 2.497 years expertise), who had previous knowledge of artificial and natural flavors through intensive learning in school and/or at work; and (iv) a group of perfumers (“perfumers”: *n* = 16; mean age, 34.063 ± 1.296 years; five male; with 9.933 ± 1.487 years expertise), who had previous knowledge of olfactory compounds for designing new fragrances through intensive learning in school and/or at work.

### ODORANTS

**FIGURE 1:**
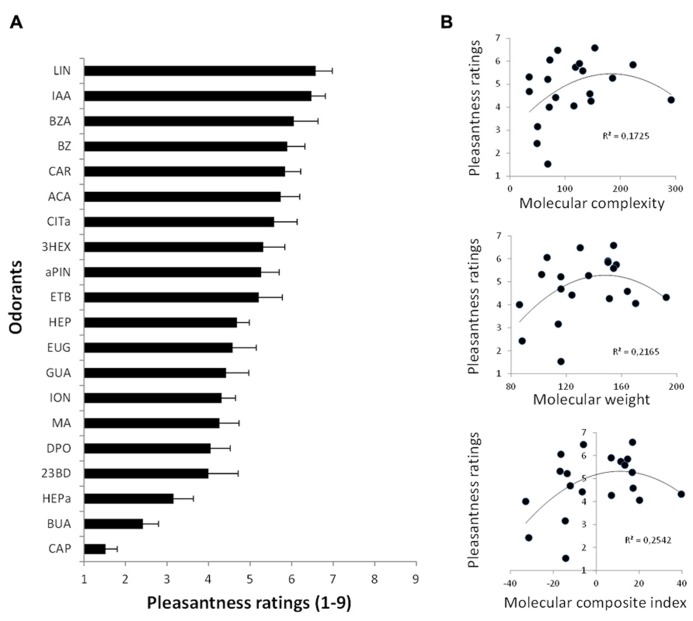
**Pilot study.** **(A)** The tested odorants cover a large range of pleasantness ratings. **(B)** Odorant molecules covered a full range of structural complexity (complexity index was collected from PubChem, one of the largest databases of chemical molecules – http://pubchem.ncbi.nlm.nih.gov/; see also Kermen et al., 2011 for details), of molecular weight and of physicochemical properties (molecular composite index based on a principal component analysis with physicochemical data obtained from Dragon^®^ software).

Twenty odorants covering a wide range of hedonic valence were used. [Odor code: compound ID; *v/v* concentration in mineral oil, as used by Kermen et al. (2011)]: 3-hexanol (3HEX: 12178; 0.076), heptanol (HEP: 8129; 0.911), butyric acid (BUA: 6590; 0.098), heptanal (HEPa: 8130; 0.075), ethyl butyrate (ETB: 7762; 0.012), caproic acid (CAP: 8892; 3.631), 2′3-butane-di-one (23BD: 650; 0.003), benzaldehyde (BZ: 240; 0.154), guaiacol (GUA: 460; 2.087), isoamylacetate (IAA: 31276; 0.032), diphenyl oxide (DPO: 7583; 13.552), allyl caproate (ACA: 31266; 0.553), benzyl acetate (BZA: 8785; 1.467), citronellal (CITa: 7794; 1.271), eugenol (EUG: 3314; 13.122), methyl anthranilate (MA: 8635; 12.653), linalol (LIN: 6549; 2.164), alpha-pinene (aPIN: 6654; 0.099), D-carvone (CAR: 16724; 1.924), and beta-ionone (ION: 638014; 30.604). To further examine the hedonic assessment of each of these odors, a pilot experiment was conducted in healthy subjects (*n* = 19; mean age, 19.47 ± 0.207 years; 13 male) who rated the pleasantness of each stimulus on a scale from 1 (not at all pleasant) to 9 (very pleasant). Results revealed that the stimuli did indeed cover a wide range of affective evaluation, from the most unpleasant to the most pleasant (**Figure 1A**). Moreover, it was also ensured that the odorants covered the entire physicochemical olfactory space by including molecules with a full range of molecular weight and structural complexity (**Figure 1B**). All odorants were diluted in mineral oil so as to achieve an approximate gas-phase partial pressure of 1 Pa.

### EXPERIMENTAL PROCEDURE

The experimental procedure was explained in great detail to the subjects, who provided written consent prior to participation. The study was conducted according to the Declaration of Helsinki and was approved by the local ethics committee of Lyon.

After providing written informed consent, subjects started the experiment. Odorants were presented in 15-ml flasks (opening diameter, 1.7 cm; height, 5.8 cm; filled with 5 ml), absorbed on a scentless polypropylene fabric (3 × 7 cm; 3M, Valley, NE, USA) to optimize evaporation and air/oil partitioning.

The experimenter presented the odorant vial 1 cm below the subject’s nose and subjects were instructed to sniff at each presentation of a vial then rate odor intensity and pleasantness on a scale from 1 (not at all intense/ pleasant) to 9 (very intense/ pleasant). Although the two ratings were performed in the same perceptual task, participants were asked to first complete the intensity judgment that refers more to the stimulus itself (i.e., concentration).

Once odor ratings were completed, participants were asked to verbalize on each odor by describing it as precisely as possible. The instructions given to the subjects were as follows: “You are going to smell several odors one after the other. Your task will be to sniff each vial and then to rate how intense and pleasant the smell was. To give your estimates, you will rate each odorant on a scale from 1 (not at all intense/ pleasant) to 9 (very intense/ pleasant). Then, after rating each odor, you will have to describe the smell as precisely as possible.” Odorants were presented every 45 s. In order to habituate the subjects to the experimental setting, a training session was carried out with a sequence of 1–3 empty flasks.

### DATA ANALYSIS

#### Partitioning the odorant data set into two groups of pleasant and unpleasant odors

A cluster analysis (using k-means partitioning) was used to separate the odorant sample into two groups of pleasant and unpleasant odors. Here, all pleasantness ratings data from all odorants and all subjects (from the four groups) were considered. This analysis revealed that the ten most unpleasant were CAP, BUA, HEPa, DPO, 23BD, HEP, MA, GUA, EUG, and ETB and the ten most pleasant odors were CITa, 3HEX, ION, aPIN, ACA, CAR, IAA, BZ, BZA, and LIN. It is noteworthy that the pleasantness scores of the 20 odorants in the main study correlated positively with those obtained in the pilot study (*r* = 0.92, *p* < 0.0001).

#### Verbal description of pleasant and unpleasant odors

**FIGURE 2:**
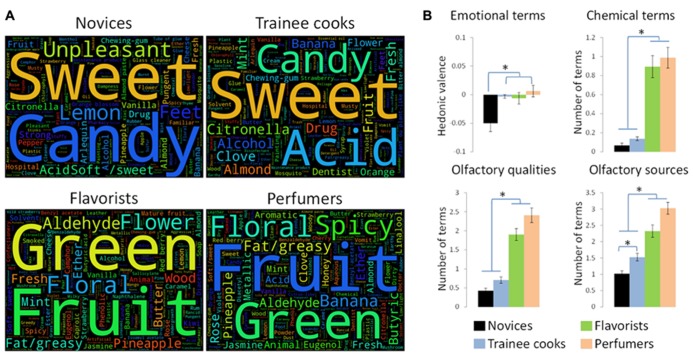
**Verbal descriptions of odors in untrained subjects, trainee cooks, flavorists, and perfumers.** **(A)** Whereas novice (untrained) subjects used more often sources and negative emotional terms to describe smells, experts used more technical terms when asked to verbalize about odors. **(B)** Novices used more emotional terms than trainee cooks, flavorists and perfumers and these terms are more often negatives. The number of chemical terms and olfactory qualities to describe odors is significantly higher in flavorists and perfumers than in novices and trainee cooks. Further, novices used less odor source references than trainee cooks, flavorists and perfumers and trainee cooks used less odor source references than flavorists and perfumers. *p < 0.05 (corrected for multiple comparisons).

To illustrate the verbal descriptions provided by the four groups, we considered the descriptions of each individual (in a given group) by counting the number of times a word was used. Thus, for each group, a table including all words and their occurrences was set. These four tables were then expressed graphically (https://github.com/amueller/word_cloud; **Figure 2A**). Afterward, to analyze each subject’s olfactory description, the 20 verbalizations produced by each subject (for the 20 odorants) were processed by exploratory lexical analysis, first counting references to pleasantness (e.g., “pleasant,” “unpleasant”). Here, a mark of “-1” was attributed for unpleasant labels, and a mark “+1” was used for pleasant labels. Second, three types of references were considered: (1) references to an odor source (e.g., “flower”), (2) references to an olfactory quality [e.g., “woody,” Chastrette et al. (1988) being used to determine whether a term was an olfactory quality], and (3) references to chemical terminology (e.g., “beta ionone”).

#### Statistical analyses

Perceptual ratings and verbal data were analyzed using a 4 × 2 ANOVA using “group” (novices, trainee cooks, flavorists, perfumers) as a between-subjects factor and “hedonic valence” (unpleasant, pleasant) as a within-subject factor. If significant effects of “group” or “hedonic valence” or a significant “group”*“hedonic valence” interaction were observed, the analysis was followed by Bonferroni tests to allow for multiple statistical comparisons.

## RESULTS

### PERCEPTUAL RATING TASK

Because the four groups had heterogeneous distributions in terms of age and gender, as a control analysis, we first explored whether age correlated with hedonic appreciation in each pleasantness category (independent of learning group). Results revealed no significant relationship between hedonic appreciation of (i) pleasant odors and age (*r* = 0.007, *p* > 0.05) and, (ii) unpleasant odors and age (*r* = 0.056, *p* > 0.05). Moreover, gender did not influence hedonic appreciation of pleasant odors [*F*(1,62) = 0.049, *p* > 0.05] and unpleasant odors [*F*(1,62) = 0.175, *p* > 0.05].

Statistical analysis of pleasantness ratings revealed a significant effect of hedonic valence [*F*(1,60) = 267.171, *p* < 0.0001; pleasant odors being rated as more pleasant than unpleasant odors; mean ± SEM: unpleasant odors, 3.86 ± 0.10; pleasant odors, 5.54 ± 0.09]. In addition, a significant effect of groups was noted [*F*(3,60) = 3.416, p < 0.03], but paired comparisons revealed no significant difference between the four groups (*p* > 0.05 in all cases; **Table 1**).

**Table 1:** Pleasantness ratings of pleasant and unpleasant odors in novice (untrained) subjects, trainee cooks, flavorists, and perfumers.

	Unpleasant odors		Pleasant odors	
	Mean	SEM	Mean	SEM
Novices	3.63	0.13	5.58	0.17
Trainee cooks	3.66	0.18	5.41	0.19
Flavorists	4.55	0.24	5.76	0.21
Perfumers	3.62	0.19	5.45	0.22

Regarding intensity ratings, a significant effect of hedonic valence was observed [*F*(2,120) = 17.008, *p* < 0.0001; pleasant odors being rated as less intense than unpleasant odors; mean ± SEM: unpleasant odors, 6.59 ± 0.10; pleasant odors, 5.75 ± 0.10].

This effect was accompanied by a significant effect of groups [*F*(3,60) = 4.045, *p* < 0.02] and a significant groups*hedonic valence interaction [*F*(3,60) = 6.108, *p* < 0.002]. The effect of groups reflected that odors were rated as significantly more intense by perfumers than novices (*p* < 0.03) (**Table 2**). The significant group*hedonic valence interaction reflected that unpleasant odors were rated more intense than pleasant odors in novices (*p* < 0.006), trainee cooks (*p* < 0.0001), flavorists (*p* < 0.005), and perfumers (*p* < 0.0001).

**Table 2:** Intensity ratings of pleasant and unpleasant odors in novice (untrained) subjects, trainee cooks, flavorists, and perfumers.

	Unpleasant odors		Pleasant odors	
	Mean	SEM	Mean	SEM
Novices	6.21	0.19	5.44	0.23
Trainee cooks	6.19	0.23	5.64	0.22
Flavorists	6.73	0.16	6.09	0.20
Perfumers	7.25	0.13	5.83	0.18

### VERBAL DESCRIPTION TASK

A descriptive analysis performed on the verbal data revealed differences between groups regarding their odor description. Whereas novices seems to use specific sources (e.g., “feet,” “candy”) and “emotional” words (e.g., “unpleasant”), flavorists and perfumers describe smells using more technical terms (chemical terminology and references to olfactory qualities and sources; **Figure 2A**).

Specifically, regarding emotional terms, a significant effect of hedonic valence was observed reflecting that unpleasant odors were described using more negative emotional words than pleasant odors [mean ± SEM: unpleasant odors, -0.033 ± 0.011; pleasant odors, 0.006 ± 0.009; *F*(3,60) = 5.958, *p* < 0.002]. Moreover, a significant effect of group was observed [*F*(1,60] = 5.306, *p* < 0.03], reflecting that novices used more negative emotional words to describe odors than trainee cooks (*p* < 0.03) and perfumers (*p* < 0.02; **Figure 2B**). It is worth noting that the difference between novices and flavorists was significant (*p* = 0.0187) but did not survive multiple comparisons.

For chemical terms, a significant effect of group was observed [*F*(3,60) = 36.353, *p* < 0.0001], reflecting the fact that perfumers and flavorists used more chemical terms than novices and trainee cooks (*p* < 0.0001). No significant differences were observed between perfumers and flavorists (*p* > 0.05) or between novices and trainee cooks (*p* > 0.05; **Figure 2B**).

With regard to olfactory qualities, a significant effect of group was likewise observed [*F*(3,60) = 48.818, *p* < 0.0001]: perfumers and flavorists used more olfactory quality terms than novices and trainee cooks (*p* < 0.005). No significant differences were observed between perfumers and flavorists (*p* > 0.05) or between novices and trainee cooks (*p* > 0.05; **Figure 2B**). In addition, a significant effect of hedonic valence was observed [mean ± SEM: unpleasant odors, 1.30 ± 0.11; pleasant odors, 1.41 ± 0.13; *F*(1,60) = 5.877, *p* < 0.02] reflecting that pleasant odors were described using more olfactory qualities than unpleasant odors.

Finally, regarding references to odor sources, a significant effect of group was also observed [*F*(2,60) = 34.622, *p* < 0.0001]: (i) novices used fewer odor source references than trainee cooks, flavorists and perfumers (*p* < 0.05); and (ii) trainee cooks used fewer odor source references than flavorists and perfumers. No significant differences were observed between perfumers and flavorists (*p* > 0.05; **Figure 2B**). Apart from main effect of group, a significant effect of hedonic valence was observed reflecting the fact that pleasant odors were described using more odor source references than unpleasant odors [mean ± SEM: unpleasant odors, 1.84 ± 0.11; pleasant odors, 2.1 ± 0.13; *F*(1,60) = 20.610, *p* < 0.0001].This latter finding corroborates previous results in the field showing a negative correlation between the number of olfactory qualities and odor unpleasantness: odorants that evoked few sources and qualities were also perceived as more unpleasant (Kermen et al., 2011).

## DISCUSSION

The main question addressed by the present investigation concerned the effect of expertise on verbal descriptions and perceptual assessments of pleasant and unpleasant odors. It was assumed that flavorists and perfumers should rate pleasant odors as less pleasant, and unpleasant odors as less unpleasant than non-experts. Moreover, on a descriptive level, whereas flavorists and perfumers were expected to use chemical and odor terminology without referring to odor hedonics, novices were expected to accompany their odor descriptions by references to pleasantness.

An important finding of the present study is that, in contrast to our expectations, hedonic perceptual ratings of unpleasant and pleasant odors was not affected by expertise: novices, trainee cooks, flavorists and perfumers rated similarly unpleasant odors on the one hand and pleasant odors on the other hand. As was shown in wine tasting (Valentin and Chollet, 2000) where experts and naïve subjects do not significantly differ in perceptual similarity judgment, the present study suggests that experts in olfaction are able to discriminate and/or categorize odors on the basis of their hedonic valence. However, although this is true at an evaluative or perceptual level (pleasantness ratings), verbal data suggest that experts describe and conceptualize odors with few references to pleasantness: a result of interest of our study was the low number of references to pleasantness in the verbal descriptions of experts, whereas novices used hedonic terms to describe odors (especially words with negative connotation). These results are in line with the literature in the field suggesting that experts in olfaction avoid references to odor hedonic valence (Yoshida, 1964; Ehrlichman and Bastone, 1992; Holley, 2002).

An interpretation of the discrepancy between an expert’s ability to use less references to unpleasantness than controls vs. his actual perceptual hedonic appreciation of unpleasant (and pleasant) odors which remains the same, could be that on a perceptual level, hedonic valence and especially its negative side, represents the basic level of odor categorization for any perceiver, independent of his/her expertise. This affective perception would occur quickly and unwittingly. In accordance with the above, autonomic responses to unpleasant odors occur implicitly when subjects are not given any particular instruction (Bensafi et al., 2002b), and response times are significantly shorter for unpleasant than for pleasant odors (Bensafi et al., 2003b). These results seem to indicate a “quick and dirty” pathway, fast-tracking decision for bad odors. Brain imaging studies also show that pleasant and unpleasant odors activate different neural networks (Zald and Pardo, 1997; Gottfried et al., 2002a; Anderson et al., 2003; Rolls et al., 2003; Royet et al., 2003; de Araujo et al., 2005; Bensafi et al., 2008). Taken together, these results support the hypothesis that only a rudimentary level of processing is necessary to hedonically pre-process odors, and that this pre-processing takes place when perceivers do not attend to any other specific feature of the odorant stimulus, whatever the expertise level. However, when experts are engaged in a verbal task requiring subtle discrimination and description, they process the same odors more deeply on a lexico-semantic level, with few hedonic references.

On a lexical level, verbal descriptions in relation to smells were significantly longer in experts than untrained subjects, confirming expectations regarding experts’ explicit knowledge. Previous studies described the language of experts (perfumers and flavorists) as richer, more proficient, precise, expressive and/or consistent (Bende and Nordin, 1997; Parr et al., 2002). In line with this, the linguistic-based criteria used here showed that experts’ verbal skills were characterized by the use of chemical names and terms referring to odor qualities and sources. Moreover, trainee cooks used more odor source references than novices, suggesting that daily exposure to odor sources (food sources in this case, without explicit olfactory associative learning) can increase the verbal ability to describe smells.

Lack of verbal resources in odor processing is a characteristic of untrained subjects. Indeed, it is a common experience to like (or dislike) a specific odor, and to be quite sure of recognizing it even if no name can be put on it: this so-called ‘tip of the nose phenomenon’ highlights implicit knowledge of odors, despite failure to name them. This interaction between language and olfaction can be seen in the development of olfactory function: whereas a 3-year-old child learns to name colors, odor naming is mostly developed through autonomous learning (Rouby and Sicard, 1997) and expressed in terms of idiosyncratic experience (Engen, 1987). On the contrary, expert verbal skills in our study were characterized by the use of domain-specific terminology, with very few references to pleasantness. These differences between experts and novices reflect the effect of learning for flavorists and perfumers since their olfactory education includes learning of chemical names and olfactory qualities with adjectives (see the “the field of odors,” Jaubert et al., 1987; Jaubert, 1995). For example, in perfumers and flavorists particularly, the creation of fragrances and flavors involves recognizing hundreds of odorants and memorizing the effects of their combinations. Reports indicate that perfumers are better able to imagine odors (Gilbert et al., 1998; see also Rinck et al., 2009) and can routinely group odors in classes, from 18 (Rimmel, 1895) to 88 (Arctander, 1969; Chastrette et al., 1988). For perfumers, these classes usually contain further sub-classes (Roudnitska, 1991; Ellena, 2007). Moreover, notions such as “notes,” “faces,” and “sub-tones” are used in perfumery to represent odors (Ellena, 2007). Experts, through such continuous repetitive olfactory training, can communicate their perception using verbal supports which is of upmost importance in their professional practice.

Although the present study provides evidence for an influence of expertise on odor verbalization, some of the findings warrant discussion. Indeed, another particular feature of the present findings was the increased perceived intensity in perfumers. One potential explanation may be that perfumers have lower odor threshold leading to higher perceived intensity due to their past training. Unfortunately, very little information is available to confirm this hypothesis and one of the few studies that compared experts and novices on a sensory level was that of (Bende and Nordin, 1997) who showed no expertise effect on olfactory detection, rendering less likely this possibility. Another explanation may be that perceived intensity is higher for identified odors (Distel and Hudson, 2001). In this psychophysical study, the authors tested human participants with a large set of everyday odorants, and asked their subjects to rate odor pleasantness, familiarity and intensity. Results showed that all these ratings (including odor intensity) were enhanced when participants either were given the name by the experimenter or could identify the odorant source themselves. In the same line, the increase in odor intensity seen in experts of our study may be related to their better ability to describe, name and identified the odors used.

In conclusion, we showed here that expertise does not influence odor hedonic perception *per se* when the subject’s attention is focused on pleasantness: experts and novices appreciated similarly pleasant and unpleasant odors. On a verbal level, in contrast to experts, novices do not have rich lexical representations of smells, and they often use words referring to environmental odor sources accompanied by perceptually hedonic terms, often referring to unpleasantness. However, when attention is directed toward the lexical component of odor representations, experts seem to avoid references to pleasantness. These findings offer new insights into odor hedonic perception in untrained and expert populations, highlighting for the first time an influence of expertise at the verbal but not at the perceptual level of processing, providing new understanding on perceptual processing of pleasant and unpleasant odors.

## Conflict of Interest Statement

The authors declare that the research was conducted in the absence of any commercial or financial relationships that could be construed as a potential conflict of interest.

## References

[B1] Alaoui-Ismaili O. Robin O. Rada H. Dittmar A. Vernet-Maury E. (1997a). Basic emotions evoked by odorants: comparison between autonomic responses and self-evaluation. *Physiol. Behav.* 62 713–720 10.1016/S0031-9384(97)90016-0 9284489

[B2] Alaoui-Ismaili O. Vernet-Maury E. Dittmar A. Delhomme G. Chanel J. (1997b). Odor hedonics: connection with emotional response estimated by autonomic parameters. *Chem. Senses* 22 237–248 10.1093/chemse/22.3.237 9218136

[B3] Anderson A. K. Christoff K. Stappen I. Panitz D. Ghahremani D. G. Glover G. (2003). Dissociated neural representations of intensity and valence in human olfaction. *Nat. Neurosci.* 6 196–202 10.1038/nn1001 12536208

[B4] Arctander S. (1969). *Perfume and Flavor Chemicals (Aroma Chemicals)*. Montclair, NJ: Allured Publishing Corporation

[B5] Ayabe-Kanamura S. Schicker I. Laska M. Hudson R. Distel H. Kobayakawa T. (1998). Differences in perception of everyday odors: a Japanese-German cross-cultural study. *Chem. Senses* 23 31–38 10.1093/chemse/23.1.31 9530967

[B6] Barkat S. Poncelet J. Landis B. N. Rouby C. Bensafi M. (2008). Improved smell pleasantness after odor-taste associative learning in humans. *Neurosci. Lett.* 434 108–112 10.1016/j.neulet.2008.01.037 18280654

[B7] Bende M. Nordin S. (1997). Perceptual learning in olfaction: professional wine tasters versus controls. *Physiol. Behav.* 62 1065–1070 10.1016/S0031-9384(97)00251-5 9333201

[B8] Bensafi M. Iannilli E. Gerber J. Hummel T. (2008). Neural coding of stimulus concentration in the human olfactory and intranasal trigeminal systems. *Neuroscience* 154 832–838 10.1016/j.neuroscience.2008.03.079 18485604

[B9] Bensafi M. Iannilli E. Poncelet J. Seo H. S. Gerber J. Rouby C. (2012). Dissociated representations of pleasant and unpleasant olfacto-trigeminal mixtures: an FMRI study. *PLoS ONE* 7:e38358 10.1371/journal.pone.0038358 PMC3373527 22701631

[B10] Bensafi M. Rinck F. Schaal B. Rouby C. (2007). Verbal cues modulate hedonic perception of odors in 5-year-old children as well as in adults. *Chem. Senses* 32 855–862 10.1093/chemse/bjm055 17728278

[B11] Bensafi M. Rouby C. Farget V. Bertrand B. Vigouroux M. Holley A. (2002a). Autonomic nervous system responses to odours: the role of pleasantness and arousal. *Chem. Senses* 27 703–709 10.1093/chemse/27.8.703 12379594

[B12] Bensafi M. Rouby C. Farget V. Bertrand B. Vigouroux M. Holley A. (2002b). Influence of affective and cognitive judgments on autonomic parameters during inhalation of pleasant and unpleasant odors in humans. *Neurosci. Lett.* 319 162–166 10.1016/S0304-3940(01)02572-1 11834318

[B13] Bensafi M. Rouby C. Farget V. Bertrand B. Vigouroux M. Holley A. (2002c). Psychophysiological correlates of affects in human olfaction. *Neurophysiol. Clin.* 32 326–332 10.1016/S0987-7053(02)00339-8 12490330

[B14] Bensafi M. Rouby C. Farget V. Vigouroux M. Holley A. (2002d). Asymmetry of pleasant vs. unpleasant odor processing during affective judgment in humans. *Neurosci. Lett.* 328 309–313 10.1016/S0304-3940(02)00548-7 12147332

[B15] Bensafi M. Porter J. Pouliot S. Mainland J. Johnson B. Zelano C. (2003a). Olfactomotor activity during imagery mimics that during perception. *Nat. Neurosci.* 6 1142–1144 10.1038/nn1145 14566343

[B16] Bensafi M. Rouby C. Farget V. Bertrand B. Vigouroux M. Holley A. (2003b). Perceptual, affective, and cognitive judgments of odors: pleasantness and handedness effects. *Brain Cogn.* 51 270–275 10.1016/S0278-2626(03)00019-8 12727181

[B17] Berglund B. Berglund U. Engen T. Ekman G. (1973). Multidimensional analysis of twenty-one odors. *Scand. J. Psychol.* 14 131–137 10.1111/j.1467-9450.1973.tb00104.x 4705857

[B18] Brauchli P. Ruegg P. B. Etzweiler F. Zeier H. (1995). Electrocortical and autonomic alteration by administration of a pleasant and an unpleasant odor. *Chem. Senses* 20 505–515 10.1093/chemse/20.5.505 8564425

[B19] Chastrette M. Elmouaffek A. Sauvegrain P. (1988). A multidimensional statistical study of similarities between 74 notes used in perfumery. *Chem. Senses* 13 295–305 10.1093/chemse/13.2.295

[B20] Croy I. Olgun S. Joraschky P. (2011). Basic emotions elicited by odors and pictures. *Emotion* 11 1331–1335 10.1037/a0024437 21787073

[B21] de Araujo I. E. Rolls E. T. Velazco M. I. Margot C. Cayeux I. (2005). Cognitive modulation of olfactory processing. *Neuron* 46 671–679 10.1016/j.neuron.2005.04.021 15944134

[B22] Distel H. Hudson R. (2001). Judgement of odor intensity is influenced by subjects’ knowledge of the odor source. *Chem. Senses* 26 247–251 10.1093/chemse/26.3.247 11287384

[B23] Dubois D. (2000). Categories as acts of meaning: the case of categories in olfaction and audition. *Cogn. Sci. Q.* 1 35–68

[B24] Dubois D. Rouby C. (1997). Une approche de l’olfaction: du linguistique au neuronal. *Intellectica* 24 9–20

[B25] Ehrlichman H. Bastone L. (1992). “Olfaction and emotion,” in *Science of Olfaction* Serby M. Chobor K. (New York: Springer) 410–438 10.1007/978-1-4612-2836-3_15

[B26] Ehrlichman H. Brown S. Zhu J. Warrenburg S. (1995). Startle reflex modulation during exposure to pleasant and unpleasant odors. *Psychophysiology* 32 150–154 10.1111/j.1469-8986.1995.tb03306.x 7630979

[B27] Ehrlichman H. Kuhl S. B. Zhu J. Warrenburg S. (1997). Startle reflex modulation by pleasant and unpleasant odors in a between-subjects design. *Psychophysiology* 34 726–729 10.1111/j.1469-8986.1997.tb02149.x 9401428

[B28] Ellena J. (2007). *La Note Verte*. Paris: Sabine Wespieser Editeur

[B29] Engen T. (1987). Remembering odors and their names. *Am. Sci.* 75 497–503

[B30] Fernandez P. Bensafi M. Rouby C. Giboreau A. (2013). Does olfactory specific satiety take place in a natural setting? *Appetite* 60 1–4 10.1016/j.appet.2012.10.006 23079143

[B31] Gilbert A. N. Crouch M. Kemp S. E. (1998). Olfactory and visual mental imagery. *J. Ment. Imagery* 22 137–146

[B32] Godinot N. Sicard G. Dubois D. (1995). Categories, familiarity and unpleasantness of odours. *Odours VOC’s J.* 3 202–208

[B33] Gottfried J. A. Deichmann R. Winston J. S. Dolan R. J. (2002a). Functional heterogeneity in human olfactory cortex: an event-related functional magnetic resonance imaging study. *J. Neurosci.* 22 10819–10828 12486175 10.1523/JNEUROSCI.22-24-10819.2002 PMC6758422

[B34] Gottfried J. A. O’Doherty J. Dolan R. J. (2002b). Appetitive and aversive olfactory learning in humans studied using event-related functional magnetic resonance imaging. *J. Neurosci.* 22 10829–10837 12486176 10.1523/JNEUROSCI.22-24-10829.2002 PMC6758414

[B35] Harper R. (1966). On odour classification. *Int. J. Food Sci. Technol.* 1 167–176 10.1111/j.1365-2621.1966.tb01803.x

[B36] Herz R. S. (2003). The effect of verbal context on olfactory perception. *J. Exp. Psychol. Gen.* 132 595–606 10.1037/0096-3445.132.4.595 14640850

[B37] Holley A. (2002). “Cognitive aspects of olfaction in perfumer practice,” in *Olfaction, Taste and Cognition* eds Rouby C. Schaal B. Dubois D. Gervais R. Holley A. (Cambridge, NY: Cambridge University Press) 16–26 10.1017/CBO9780511546389.006

[B38] Jacob T. J. Fraser C. Wang L. Walker V O’Connor S. (2003). Psychophysical evaluation of responses to pleasant and mal-odour stimulation in human subjects; adaptation, dose response and gender differences. *Int. J. Psychophysiol.* 48 67–80 10.1016/S0167-8760(03)00020-5 12694902

[B39] Jaubert J. N. (1995). The field of odors: toward a universal language for odor relationships. *Perfumer and flavorist* 20 1–15

[B40] Jaubert J. N. Gordon G. Dore J.-C. (1987). Une organisation du champ des odeurs. *Parfums, cosmétiques, arômes* 77 53–56

[B41] Joussain P. Chakirian A. Kermen F. Rouby C. Bensafi M. (2011). Physicochemical influence on odor hedonics: where does it occur first? *Commun. Integr. Biol*. 4 563–565 10.4161/cib.4.5.15811 22046463 PMC3204129

[B42] Joussain P. Giboreau A. Fontas M. Laville M. Hummel T. Souquet P. J. (2013a). Cisplatin chemotherapy induces odor perception changes in bronchial cancer patients. *Lung Cancer* 82 168–170 10.1016/j.lungcan.2013.06.009 23896024

[B43] Joussain P. Thevenet M. Rouby C. Bensafi M. (2013b). Effect of aging on hedonic appreciation of pleasant and unpleasant odors. *PLoS ONE* 8:e61376 10.1371/journal.pone.0061376 PMC3634785 23637821

[B44] Kermen F. Chakirian A. Sezille C. Joussain P. Le Goff G. Ziessel A. (2011). Molecular complexity determines the number of olfactory notes and the pleasantness of smells. *Sci. Rep.* 1 206 10.1038/srep00206 PMC3244502 22355721

[B45] Khan R. M. Luk C. H. Flinker A. Aggarwal A. Lapid H. Haddad R. (2007). Predicting odor pleasantness from odorant structure: pleasantness as a reflection of the physical world. *J. Neurosci.* 27 10015–10023 10.1523/JNEUROSCI.1158-07.2007 17855616 PMC6672642

[B46] Mandairon N. Poncelet J. Bensafi M. Didier A. (2009). Humans and mice express similar olfactory preferences. *PLoS ONE* 4:e4209 10.1371/journal.pone.0004209 PMC2615132 19148286

[B47] Miltner W. Matjak M. Braun C. Diekmann H. Brody S. (1994). Emotional qualities of odors and their influence on the startle reflex in humans. *Psychophysiology* 31 107–110 10.1111/j.1469-8986.1994.tb01030.x 8146248

[B48] Parr W. V. Heatherbell D. White K. G. (2002). Demystifying wine expertise: olfactory threshold, perceptual skill and semantic memory in expert and novice wine judges. *Chem. Senses* 27 747–755 10.1093/chemse/27.8.747 12379599

[B49] Poncelet J. Rinck F. Ziessel A. Joussain P. Thevenet M. Rouby C. (2010). Semantic knowledge influences prewired hedonic responses to odors. *PLoS ONE* 5:e13878 10.1371/journal.pone.0013878 PMC2975635 21079734

[B50] Rimmel E. (1895). *The Base of Perfumes*. London (French translation 1990, Le livre des parfums. Paris: les éditions 1900).

[B51] Rinck F. Barkat-Defradas M. Chakirian A. Joussain P. Bourgeat F. Thevenet M. (2011). Ontogeny of odor liking during childhood and its relation to language development. *Chem. Senses* 36 83–91 10.1093/chemse/bjq101 20956736

[B52] Rinck F. Rouby C. Bensafi M. (2009). Which format for odor images? *Chem. Senses* 34 11–13 10.1093/chemse/bjn060 18854509

[B53] Rolls E. T. (2004). The functions of the orbitofrontal cortex. *Brain Cogn.* 55 11–29 10.1016/S0278-2626(03)00277-X 15134840

[B54] Rolls E. T. Kringelbach M. L De Araujo I. E. (2003). Different representations of pleasant and unpleasant odours in the human brain. *Eur. J. Neurosci.* 18 695–703 10.1046/j.1460-9568.2003.02779.x 12911766

[B55] Rouby C. Bourgeat F. Rinck F. Poncelet J. Bensafi M. (2009). Perceptual and sensorimotor differences between “good” and “poor” olfactory mental imagers. *Ann. N. Y. Acad. Sci.* 1170 333–337 10.1111/j.1749-6632.2009.03915.x 19686156

[B56] Rouby C. Sicard G. (1997). “Des catégories d’odeurs,” in *Catégorisation et cognition : de la perception au discours* ed. Dubois D. (Paris: Edition Kimé) 59–81

[B57] Roudnitska E. (1991). “The art of perfumery,” in *Perfumes Art, Science and Technology* eds Müller P. M. Lamparsky D. (London: Elsevier) 3–48

[B58] Royet J. P. Plailly J. Delon-Martin C. Kareken D. A. Segebarth C. (2003). fMRI of emotional responses to odors: influence of hedonic valence and judgment, handedness, and gender. *Neuroimage* 20 713–728 10.1016/S1053-8119(03)00388-4 14568446

[B59] Schiffman S. (1977). Food recognition by the elderly. *J. Gerontol.* 32 586–592 10.1093/geronj/32.5.586 886165

[B60] Schiffman S. S. (1974). Physicochemical correlates of olfactory quality. *Science* 185 112–117 10.1126/science.185.4146.112 4834219

[B61] Valentin D. Chollet S. (2000). Le degré d’expertise a-t-il une influence sur la perception olfactive? Quelques éléments de réponse dans le domaine du vin. *L’année psychologique* 100 11–36 10.3406/psy.2000.28625

[B62] Yeshurun Y. Sobel N. (2010). An odor is not worth a thousand words: from multidimensional odors to unidimensional odor objects. *Annu. Rev. Psychol.* 61, 219–241 C211–215 10.1146/annurev.psych.60.110707.163639 19958179

[B63] Yoshida M. (1964). Studies in psychometric classification of odors. *Jpn. Psychol. Res.* 6 115–124

[B64] Zald D. H. Pardo J. V. (1997). Emotion, olfaction, and the human amygdala: amygdala activation during aversive olfactory stimulation. *Proc. Natl. Acad. Sci. U.S.A.* 94 4119–4124 10.1073/pnas.94.8.4119 9108115 PMC20578

[B65] Zarzo M. (2011). Hedonic judgments of chemical compounds are correlated with molecular size. *Sensors (Basel)* 11 3667–3686 10.3390/s110403667 22163815 PMC3231300

[B66] Zucco G. M. Carassai A. Baroni M. R. Stevenson R. J. (2011). Labeling, identification, and recognition of wine-relevant odorants in expert sommeliers, intermediates, and untrained wine drinkers. *Perception* 40 598–607 10.1068/p6972 21882722

